# Advanced Interval Type-2 Fuzzy Sliding Mode Control for Robot Manipulator

**DOI:** 10.1155/2017/9640849

**Published:** 2017-02-08

**Authors:** Ji-Hwan Hwang, Young-Chang Kang, Jong-Wook Park, Dong W. Kim

**Affiliations:** ^1^Republic of Korea Naval Logistics Command, P.O. Box 602, Hyeon-dong, Jinhae-gu, Changwon-si, Gyeongsangnam-do 645-798, Republic of Korea; ^2^Department of Computer Engineering, Gachon University, Bokjeong-dong, Sujeong-gu, Seongnam-si, Gyeonggi-do 461-701, Republic of Korea; ^3^Department of Electronic Engineering, Incheon National University, Incheon 402-752, Republic of Korea; ^4^Department of Digital Electronics, Inha Technical College, 100 Inha-ro, Nam-Gu, Incheon 402-752, Republic of Korea

## Abstract

In this paper, advanced interval type-2 fuzzy sliding mode control (AIT2FSMC) for robot manipulator is proposed. The proposed AIT2FSMC is a combination of interval type-2 fuzzy system and sliding mode control. For resembling a feedback linearization (FL) control law, interval type-2 fuzzy system is designed. For compensating the approximation error between the FL control law and interval type-2 fuzzy system, sliding mode controller is designed, respectively. The tuning algorithms are derived in the sense of Lyapunov stability theorem. Two-link rigid robot manipulator with nonlinearity is used to test and the simulation results are presented to show the effectiveness of the proposed method that can control unknown system well.

## 1. Introduction

In control engineering, the design of robust controller for a class of uncertain nonlinear multiple-input multiple-output (MIMO) systems remains one of the most challenging tasks. When MIMO systems are nonlinear and uncertain, their control problem becomes more challenging.

Conventional control theory is well suited to applications, where the control inputs can be generated based on analytical model [1, 2]. Sliding mode control (SMC), which is based on the theory of variable structure systems (VSS), has been widely applied to robust control of nonlinear systems [3–5]. SMC performs well in trajectory tracking of some nonlinear systems. The SMC employs a discontinuous control law to drive the state trajectory toward a specified sliding surface and maintain its motion along the sliding surface in the state space. Hung et al. [3] have made a comprehensive survey of the VSS theory. The dynamic performance of the SMC system has been confirmed as an effective robust control approach with respect to system uncertainties and unknown disturbance when the system trajectories belong to predetermined sliding surface [4].

Although the SMC performs well in the nonlinear systems, it suffers from some difficulties. First, due to the highly coupled nonlinear and uncertain dynamics, it is generally difficult or even impossible for many physical systems to obtain accurate mathematical models. Secondly, to operate effectively in the sliding surface, the SMC requires instantaneous change of the control input without sacrificing the robustness against the model uncertainties and external disturbances. The discontinuity in the control action becomes the cause of chattering, which is undesirable in most applications [6]. In the practical implementation, the chattering may cause an unnecessarily large control signal as the system uncertainties are large and may damage system components such as actuators. Thus, the chattering has to be eliminated or alleviated as much as possible. Finally, it is difficult to directly extend the SMC design into a multiple-input multiple-output (MIMO) system, especially when the coupling among the subsystems is unknown.

During the last two decades fuzzy logic system (FLS) has been a dominant topic in intelligent systems research or control community. Because the FLS provide a systematic and efficient framework to incorporate linguistic fuzzy information from human expert, it is particularly suitable for those systems with uncertain or complex dynamics. Owing to universal approximation capability [7] of fuzzy system, many FLS schemes have been developed for handling nonlinear systems, especially in the presence of incomplete knowledge of the system [8, 9].

Some researchers applied fuzzy system to sliding mode control to improve the performance of SMC. The fuzzy sliding mode control (FSMC) forms the equivalent control of SMC. By employing the FLS, the set of linearized mathematical model can be integrated into a global model that is equivalent to the nonlinear system [10, 11].

As an extension of the well-known ordinary fuzzy set (type-1 fuzzy sets), the concept of type-2 fuzzy sets (T2FS) was first introduced by Zadeh [12]. The sets are fuzzy sets whose membership grades themselves are type-1 fuzzy sets. They are very useful in circumstances where it is difficult to determine an exact membership function for a fuzzy set. They are useful for incorporating uncertainties [13].

In this paper, we propose a novel advanced interval type-2 fuzzy sliding mode control (AIT2FSMC) for a class of uncertain nonlinear MIMO systems. To inherit the strength of these two methods, we combine IT2FLS and SMC into one methodology. The AIT2FSMC system is comprised of a fuzzy control design and a hitting control design. For resembling a feedback linearization (FL) control law, IT2 fuzzy system is designed. For compensating the approximation error between the FL control law and IT2 fuzzy system, sliding mode controller is designed, respectively.

The tuning algorithms are derived in the sense of Lyapunov stability theorem. The two-link robot manipulator is used to test the proposed method and the simulation results show the AIT2FSMC can control the unknown system well.

The organization of this paper is as follows. Problem formulation and notation are presented in Section 2. In Section 3, IT2FLS is briefly introduced.  Section 4 describes the design process and the stability analysis of AIT2FSMC. In Section 5, the simulation results are presented to show the effectiveness of the proposed control for a two-link robot manipulator. Finally, conclusions are given in Section 6.

## 2. Notation and Problem Formulation

In this section, we present the problem formulation for a class of MIMO nonlinear dynamic systems. Consider the following class of MIMO nonlinear dynamic systems:

(1) $$\begin{array}{c} y_{1}^{\left(r_{1}\right)}=f_{1}\left(\;x\;\right)+\overset{p}{\underset{j=1}{\sum }}g_{1j}\left(\;x\;\right)u_{j},\\ \vdots \\ y_{p}^{\left(r_{p}\right)}=f_{p}\left(\;x\;\right)+\overset{p}{\underset{j=1}{\sum }}g_{pj}\left(\;x\;\right)u_{j}, \end{array}$$

where $x={\left[\begin{array}{ccccccccc} y_{1} & {\dot{y}}_{1} & \cdots & y_{1}^{\left(r_{1}-1\right)} & \cdots & y_{p} & {\dot{y}}_{p} & \cdots & y_{p}^{\left(r_{p}-1\right)} \end{array}\right]}^{T}\in R^{r}$ is the fully measurable state vector and *r*_1_ + ⋯+*r*_*p*_ = *r*, $u={\left[\begin{array}{ccc} u_{1} & \cdots & u_{p} \end{array}\right]}^{T}\in R^{p}$ is the control input vector, $y={\left[\begin{array}{ccc} y_{1} & \cdots & y_{p} \end{array}\right]}^{T}\in R^{p}$ is the output vector, and *f*_*i*_(*x*), *i* = 1,…, *p* are continuous nonlinear functions, and *g*_*ij*_(*x*), *i*, *j* = 1,…, *p* are continuous nonlinear *C*^1^ functions.

Let us denote

(2) yr=y1r1⋯yprpT,Fx=f1x⋯fpxT,Gx=g11x⋯g1px⋮⋱⋮gp1x⋯gppx.

Then, system (1) can be rewritten in the following compact form:

(3) $$\begin{array}{c} y^{\left(r\right)}=F\left(\;x\;\right)+G\left(\;x\;\right)u. \end{array}$$

The control problem is to design a control law *u*(*t*) which assures that the system tracks a *p*-dimensional desired vector $y_{d}={\left[\begin{array}{cccc} y_{d1} & y_{d2} & \cdots & y_{dp} \end{array}\right]}^{T}\in R^{p}$, which belongs to a class of continuous functions on [*t*_0_, *∞*. In this paper, we make the following assumption.

**Assumption 1 .**

The matrix *G*(*x*) is positive definite; then there exists *σ*_0_ > 0, *σ*_0_ ∈ *R* such that *G*(*x*) ≥ *σ*_0_*I*_*p*_, with *I*_*p*_ being an identity matrix. In the following *σ*_0_ may be known or not.

Although this assumption restricts the considered class of MIMO nonlinear systems, many physical systems, such as robotic systems [5], fulfill the above property.

**Assumption 2 .**

The desired trajectory *y*_*di*_(*t*), *i* = 1,…, *p*, is a known bounded function of time with bounded known derivatives, and *y*_*di*_(*t*) is assumed to be *r*_*i*_-times differentiable.

Let us define the tracking error as

(4) $$\begin{array}{c} e_{1}\left(\;t\;\right)=y_{d1}\left(\;t\;\right)-y_{1}\left(\;t\;\right),\\ \vdots \\ e_{p}\left(\;t\;\right)=y_{dp}\left(\;t\;\right)-y_{p}\left(\;t\;\right), \end{array}$$

and the sliding surfaces as

(5) $$\begin{array}{c} s_{1}\left(\;t\;\right)={\left(\;\frac{d}{dt}+\lambda_{1}\;\right)}^{r_{1}-1}e_{1}\left(\;t\;\right),\;\lambda_{1}>0,\\ \vdots \\ s_{p}\left(\;t\;\right)={\left(\;\frac{d}{dt}+\lambda_{p}\;\right)}^{r_{p}-1}e_{p}\left(\;t\;\right),\;\lambda_{p}>0. \end{array}$$

The time derivatives of the sliding surfaces can be written as

(6) $$\begin{array}{c} {\dot{s}}_{1}=v_{1}-f_{1}\left(\;x\;\right)-\overset{p}{\underset{j=1}{\sum }}g_{1j}\left(\;x\;\right)u_{j},\\ \vdots \\ {\dot{s}}_{p}=v_{p}-f_{p}\left(\;x\;\right)-\overset{p}{\underset{j=1}{\sum }}g_{pj}\left(\;x\;\right)u_{j}, \end{array}$$

where *v*_1_,…, *v*_*p*_ are given as follows:

(7) $$\begin{array}{c} v_{1}=y_{d1}^{\left(r_{1}\right)}+\beta_{1,r_{1}-1}e_{1}^{\left(r_{1}-1\right)}+\cdots +\beta_{1,1}{\dot{e}}_{1},\\ \vdots \\ v_{p}=y_{dp}^{\left(r_{p}\right)}+\beta_{p,r_{p}-1}e_{p}^{\left(r_{p}-1\right)}+\cdots +\beta_{p,1}{\dot{e}}_{p}, \end{array}$$

where

(8) βi,j=ri−1!ri−j!j−1!λiri−j,i=1,…,p,  j=1,…,ri−1.

Denote

(9) st=s1t⋯sptT,vt=v1t⋯vptT.

Then, (6) can be written in the compact form

(10) $$\begin{array}{c} \dot{s}=v-F\left(\;x\;\right)-G\left(\;x\;\right)u. \end{array}$$

If the nonlinear functions *F*(*x*) and *G*(*x*) are known, one can use a sliding mode controller. When the closed loop system is in the sliding mode, it satisfies $\dot{s}=0$, and then the traditional sliding mode control law is obtained by the following equation:

(11) $$\begin{array}{c} u=u_{\text{eq}}+u_{h}=G^{-1}\left(\;x\;\right)\left[\;-F\left(\;x\;\right)+v+K_{0}\mathrm{sgn}\left(\;s\;\right)\;\right], \end{array}$$

where *u*_eq_ = *G*^−1^(*x*)[−*F*(*x*) + *v*] is an equivalent control law and *u*_*h*_ = *G*^−1^(*x*)*K*_0_sgn⁡(*s*) is a hitting control law and *K*_0_ = diag⁡[*k*_01_,…, *k*_0*p*_] with *k*_0*i*_ > 0 for *i* = 1,…, *p*. Using (10) and (11), we can obtain the following equation:

(12) $$\begin{array}{c} \dot{s}=-K_{0}\mathrm{sgn}\left(\;s\;\right). \end{array}$$

Multiplying *s*^*T*^ to (12) gives

(13) $$\begin{array}{c} s^{T}\dot{s}=-s^{T}K_{0}\mathrm{sgn}\left(\;s\;\right). \end{array}$$

Let us consider the following Lyapunov function candidate:

(14) $$\begin{array}{c} V=\frac{1}{2}s^{T}s \end{array}$$

whose time derivative is given by

(15) $$\begin{array}{c} \dot{V}=s^{T}\dot{s}. \end{array}$$

With (13), (15) can be reexpressed as

(16) $$\begin{array}{c} \dot{V}=-s^{T}K_{0}\mathrm{sgn}\left(\;s\;\right)=-\overset{p}{\underset{i=1}{\sum }}k_{0i}\left|\;s_{i}\;\right|<0 \end{array}$$

which implies that *s*_*i*_(*t*) → 0 as *t* → *∞*. Therefore, *e*_*i*_(*t*) and all its derivatives up to *r*_*i*_ − 1 converge to zero [5].

According to the above analysis, the control law (11) is easily obtained if the nonlinear functions *f*_*i*_(*x*) and *g*_*ij*_(*x*) are known. However, in this paper, these nonlinear functions are assumed to be unknown, so the above design method cannot be applied directly.

## 3. Interval Type-2 Fuzzy Logic System

The theory and design of interval type-2 fuzzy logic systems (FLS) are presented well in [13–15]. The brief description of the interval type-2 FLS is depicted here. Detailed descriptions can be found in [13–15]. In particular, refer to [13, 15] for more notations and calculations of type-2 fuzzy logic equations.

A T2FS in the universal set *X* is denoted as $\tilde{A}$ which is characterized by a type-2 membership function $u_{\tilde{A}}(x)$ in (17). $u_{\tilde{A}}(x)$ can be referred to as a secondary membership function (MF) or also referred to as secondary set, which is a type-1 set in [0,1]. In (17) *f*_*x*_(*u*) is a secondary grade, which is the amplitude of a secondary MF; that is, 0 ≤ *f*_*x*_(*u*) ≤ 1. The domain of a secondary MF is called the primary membership of *x*. In (17), *J*_*x*_ is the primary membership of *x*, where *u* ∈ *J*_*x*_⊆[0,1] for ∀*x* ∈ *X*; *u* is a fuzzy set in [0,1], rather than a crisp point in [0,1].

(17) A~=∫x∈XuA~xx=∫x∈X∫u∈Jxfxu/uxJx⊆0,1.

When *f*_*x*_(*u*) = 1, ∀*u* ∈ *J*_*x*_⊆[0,1], then the secondary MFs are interval sets such that $u_{\tilde{A}}\left(x\right)$ in (17) can be called an interval type-2 MF [13]. Therefore, T2FS $\tilde{A}$ can be rewritten as

(18) $$\begin{array}{c} \tilde{A}=\overset{}{\underset{x\in X}{\int }}\frac{u_{\tilde{A}}\left(x\right)}{x}=\overset{}{\underset{x\in X}{\int }}\frac{\left[\int_{u\in J_{x}}^{}1/u\right]}{x}\;J_{x}\subseteq \left[0,1\right]. \end{array}$$

**Figure 1:**
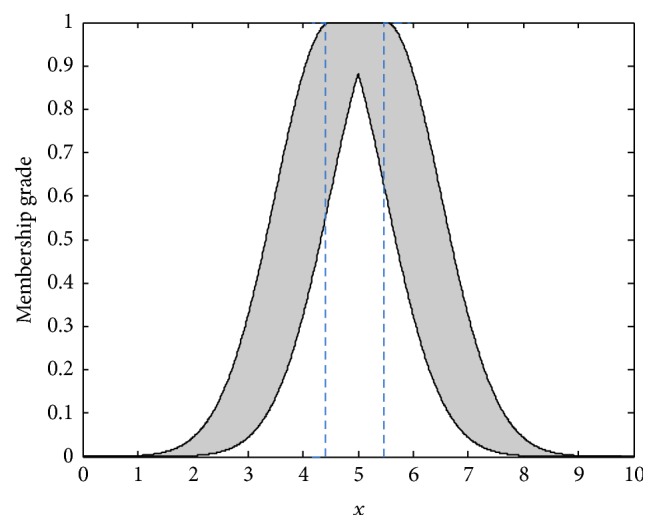
Interval type-2 Gaussian fuzzy set with uncertain mean.

Also, a Gaussian primary MF with uncertain mean and fixed standard deviation having an interval type-2 secondary MF can be called an interval type-2 Gaussian MF. A 2D interval type-2 Gaussian MF with an uncertain mean in [*m*_1_, *m*_2_] and a fixed standard deviation *σ* is shown in Figure 1. It can be expressed as

(19) $$\begin{array}{c} u_{\tilde{A}}\left(\;x\;\right)=\exp\left[\;-\frac{1}{2}{\left(\;\frac{x-m}{\sigma }\;\right)}^{2}\;\right],\;m\in \left[m_{1},m_{2}\right]. \end{array}$$

It is obvious that the T2FS in a region is called a footprint of uncertainty (FOU) and bounded by an upper MF and a lower MF [13], which are denoted as ${\overset{-}{u}}_{\tilde{A}}\left(x\right)$ and ${\underline{u}}_{\tilde{A}}\left(x\right)$, respectively. Both of them are type-1 MFs. Hence, (18) can be reexpressed as

(20) $$\begin{array}{c} \tilde{A}=\overset{}{\underset{x\in X}{\int }}\frac{\left[\int_{\mu \in \left[{\underline{u}}_{\tilde{A}}\left(x\right),{\overset{-}{u}}_{\tilde{A}}\left(x\right)\right]}^{}1/u\right]}{x}. \end{array}$$

**Figure 2:**
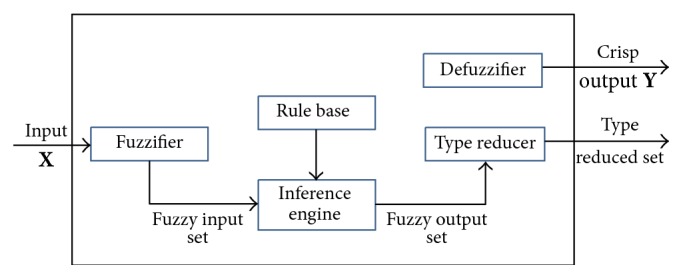
The structure of T2FLS.

A T2FLS is very similar to a T1FLS as shown in Figure 2 [13], the major structure difference being that the defuzzifier block of a T1FLS is replaced by the output processing block in a T2FLS, which consists of type-reduction followed by defuzzification.

There are five main parts in a T2FLS: fuzzifier, rule base, inference engine, type-reducer, and defuzzifier. A T2FLS is a mapping *f* : *R*^*p*^ → *R*^1^. After fuzzification, fuzzy inference, type-reduction, and defuzzification, a crisp output can be obtained.

Consider a T2FLS having *p* inputs *x*_1_ ∈ *X*_1_,…, *x*_*p*_ ∈ *X*_*p*_ and one output *y* ∈ *Y*. The type-2 fuzzy rule base consists of a collection of IF-THEN rules. We assume there are *M* rules and the rule of a type-2 relation between the input space *X*_1_ × *X*_2_ × ⋯×*X*_*p*_ and the output space *Y* can be expressed as

(21) Rule  i:  IF x1  is  F~1i  and…and  xp  is  F~pi,THEN y  is  G~i,i=1,2,…,M,

where ${\tilde{F}}_{j}^{i}$s are antecedent T2FSs (*j* = 1,2,…, *p*) and ${\tilde{G}}^{i}$s are consequent T2FSs.

The inference engine combines rules and gives a mapping from input T2FSs to output T2FSs. To achieve this process, we have to compute unions and intersections of type-2 set, as well as compositions of type-2 relations. The output of inference engine block is a type-2 set. By using the extension principle of type-1 defuzzification method, type-reduction takes us from type-2 output sets of the FLS to a type-1 set called the “type-reduced set.” This set may then be defuzzified to obtain a single crisp value.

In Figure 2, we only consider singleton input fuzzification throughout this paper. Similar to T1FLS, the firing strength *F*^*i*^ in (22) can be obtained by following inference process:

(22) $$\begin{array}{c} F^{i}=\underset{x\in X}{∐}\left[\;\overset{p}{\underset{k=1}{\prod }}u_{{\tilde{F}}_{k}^{i}}\left(\;x_{k}\;\right)\;\right], \end{array}$$

where ∏  is the meet operation and ∐  is the join operation [13].

For Gaussian IT2FS as shown in Figure 1, the upper MF is a subset that has the maximum membership grade and the lower MF is a subset that has the minimum membership grade. The join operation in (22) leads to joining the result from meet operations, which is using maximum value. The result of join operation can be an interval type-1 set [13] as

(23) $$\begin{array}{c} F^{i}={\left[\;\begin{array}{cc} {\underline{f}}^{i} & {\overset{-}{f}}^{i} \end{array}\;\right]}^{T}, \end{array}$$

where

(24) f_i=u_F~1ix1∗⋯∗u_F~pixp,f−i=u−F~1ix1∗⋯∗u−F~pixp.

There are many kinds of type-reduction, such as centroid, height, modified weight, and center-of-sets [13]. The center-of-sets type-reduction will be used in this paper and can be expressed as

(25) Ycosx=yl,yr=∫y1∈yl1,yr1⋯·∫yM∈ylM,yrM∫f1∈f_1,f−1⋯·∫fM∈f_M,f−M1∑i=1Mfiyi/∑i=1Mfi,

where *Y*_cos_ is the interval set determined by two end points *y*_l_ and *y*_*r*_, and firing strengths $f^{i}\in F^{i}=\left[{\underline{f}}^{i},{\overset{-}{f}}^{i}\right]$. The interval set $\left[\begin{array}{cc} y_{l} & y_{r} \end{array}\right]$ should be computed or set first before the computation of *Y*_cos_(**x**). For any value *y* ∈ *Y*_cos_, *y* can be expressed as

(26) $$\begin{array}{c} y=\frac{\sum_{i=1}^{M}f^{i}y^{i}}{\sum_{i=1}^{M}f^{i}}, \end{array}$$

where *y* is a monotonic increasing function with respect to *y*^*i*^. Also, *y*_*l*_ in (25) is the minimum associated only with *y*_*l*_^*i*^, and *y*_*r*_ in (25) is the maximum associated only with *y*_*r*_^*i*^. Note that *y*_*l*_ and *y*_*r*_ depend only on mixture of ${\underline{f}}^{i}$ or ${\overset{-}{f}}^{i}$ values. Hence, left-most point *y*_*l*_ and right-most point *y*_*r*_ can be expressed as [13]

(27) yl=∑i=1Mfliyli∑i=1Mfli,yr=∑i=1Mfriyri∑i=1Mfri.

For illustrative purpose, we briefly provide the computation procedure for *y*_*r*_. Without loss of generality, assume *y*_*r*_^*i*^s are arranged in ascending order; that is, *y*_*r*_^1^ ≤ *y*_*r*_^2^ ⋯ ≤*y*_1_^*M*^.

**Step 1 .**

Compute *y*_*r*_ in (27) by initially using $f_{r}^{i}=\left({\underline{f}}^{i}+{\overset{-}{f}}^{i}\right)/2$ for *i* = 1,…, *M*, where ${\underline{f}}^{i}$ and ${\overset{-}{f}}^{i}$ are precomputed by (24); and let *y*_*r*_′ = *y*_*r*_.

**Step 2 .**

Find *R*  (1 ≤ *R* ≤ *M* − 1) such that *y*_*r*_^*R*^ ≤ *y*_*r*_′ ≤ *y*_*r*_^*R*+1^.

**Step 3 .**

Compute *y*_*r*_ in (27) with $f_{r}^{i}={\underline{f}}^{i}$ for *i* ≤ *R* and $f_{r}^{i}={\overset{-}{f}}^{i}$ for *i* > *R*, and let *y*_*r*_′′ ≡ *y*_*r*_.

**Step 4 .**

If *y*_*r*_′′ ≠ *y*_*r*_′, then go to Step 5. If *y*_*r*_′′ = *y*_*r*_′, then stop and set *y*_*r*_′′ = *y*_*r*_.

**Step 5 .**

Set *y*_*r*_′ equal to *y*_*r*_′′, and return to Step 2.

This algorithm decides the point to separate two sides by the number *R*, one side using lower firing strengths ${\underline{f}}^{r}$'s and another side using upper firing strengths ${\overset{-}{f}}^{r}$'s. Hence, *y*_*r*_ in (27) can be reexpressed as

(28) yryrf_1,…,f_R,f−R+1,…,f−M,yr1,…,yrM=∑i=1Rf_iyri+∑i=R+1Mf−iyri∑i=1Rf_i+∑i=R+1Mf−i.

The procedure to compute *y*_*l*_ is similar to computing *y*_*r*_. In Step 2, it only needs to find *L*  (1 ≤ *L* ≤ *M* − 1), such that *y*_*l*_^*L*^ ≤ *y*_*l*_′ ≤ *y*_*l*_^*L*+1^. In Step 3, let $f_{l}^{i}={\overset{-}{f}}^{i}$ for *i* ≤ *L*, and $f_{r}^{i}={\underline{f}}^{i}$ for *i* > *L*. The *y*_*l*_ in (27) can be also rewritten as

(29) ylylf−1,…,f−L,f_L+1,…,f_M,yl1,…,ylM=∑i=1Lf−iyli+∑i=L+1Mf_iyli∑i=1Lf−i+∑i=L+1Mf_i.

The defuzzified crisp output from an IT2FLS is the average of

(30) $$\begin{array}{c} y\left(\;x\;\right)=\frac{y_{l}+y_{r}}{2}. \end{array}$$

## 4. Interval Type-2 Fuzzy Sliding Mode Control

In this section, we propose an adaptive interval type-2 fuzzy sliding mode controller (AIT2FSMC) for nonlinear unknown MIMO systems. Due to unknown functions *f*_*i*_(*x*) and *g*_*ij*_(*x*) in our problem, it is impossible to obtain the control law (11). We use the interval type-2 fuzzy system to approximate unknown functions *f*_*i*_(*x*) and *g*_*ij*_(*x*). First, let the nonlinear functions *f*_*i*_(*x*) and *g*_*ij*_(*x*) be approximated, over a compact set *D*_*X*_, by interval type-2 fuzzy systems as follows:

(31) f^ix,α~fi=ξfiTxα~fi,i=1,…,p,g^ijx,α~gij=ξgijTxα~gij,i,j=1,…,p,

where *ξ*_*fi*_(*x*) and *ξ*_*gij*_(*x*) are fuzzy basis vectors fixed by the designer and ${\tilde{\alpha }}_{fi}$ and ${\tilde{\alpha }}_{gij}$ are the corresponding adjustable parameter vectors of each interval type-2 fuzzy system.

Let us define

(32) α~fi∗=arg minα~fisupx∈DXfix−f^ix,α~fi,α~gij∗=arg minα~gijsupx∈DXgijx−g^ijx,α~gij

as the optimal parameters of ${\tilde{\alpha }}_{fi}$ and ${\tilde{\alpha }}_{gij}$, respectively. Notice that optimal parameters ${\tilde{\alpha }}_{fi}^{*}$ and ${\tilde{\alpha }}_{gij}^{*}$ are artificial constant quantities introduced only for analytical purpose, and their values are not needed for the implementation. Define

(33) α~−fi=α~fi∗−α~fi,α~−gij=α~gij∗−α~gij

as the parameter estimation errors, and

(34) εfix=fix−f^ix,α~fi∗,εgijx=gijx−g^ijx,α~gij∗

as the minimum fuzzy approximation errors, which correspond to approximation errors obtained when optimal parameters are used.

In this paper, we assume that the used interval type-2 fuzzy systems do not infringe the universal approximation property on the compact set *D*_*X*_, which is assumed large enough so that state variables remain within *D*_*X*_ under closed loop control. Therefore, it is reasonable to assume that the minimum approximation errors are bounded for all *x* ∈ *D*_*X*_; that is,

(35) εfix≤ε−fi,εgijx≤ε−gij,∀x∈DX,

where ${\overset{-}{\varepsilon }}_{fi}$ and ${\overset{-}{\varepsilon }}_{gij}$ are given constants.

Denote

(36) F^x,α~f=f^1x,α~f1⋯f^px,α~fpT,G^x,α~g=g^11x,α~g11⋯g^1px,α~g1p⋮⋱⋮g^p1x,α~gp1⋯g^ppx,α~gpp,εfx=εf1x⋯εfpxT,εgx=εg11x⋯εg1px⋮⋱⋮εgp1x⋯εgppx,ε−f=ε−f1⋯ε−fpT,ε−g=ε−g11⋯ε−g1p⋮⋱⋮ε−gp1⋯ε−gpp.

From the above analysis, we have

(37) Fx−F^x,α~f=F^x,α~f∗−F^x,α~f+εfx,Gx−G^x,α~g=G^x,α~g∗−G^x,α~g+εgx.

Now, let us consider the control law, *u* = *u*_*s*_, where *u*_*s*_ is a sliding mode control term [4] defined as

(38) $$\begin{array}{c} u_{s}={\hat{G}}^{-1}\left(\;x,{\tilde{\alpha }}_{g}\;\right)\left[\;-\hat{F}\left(\;x,{\tilde{\alpha }}_{f}\;\right)+v+K_{0}\mathrm{sgn}\left(\;s\;\right)\;\right]. \end{array}$$

The above control term results from (11) by using the adaptive interval type-2 fuzzy approximation $\hat{F}\left(x,{\tilde{\alpha }}_{f}\right)$ and $\hat{G}\left(x,{\tilde{\alpha }}_{g}\right)$ instead of actual functions *F*(*x*) and *G*(*x*), respectively.

The sliding mode control law (38) is not well-defined when the estimated matrix $\hat{G}\left(x,{\tilde{\alpha }}_{g}\right)$ is singular. The matrix $\hat{G}\left(x,{\tilde{\alpha }}_{g}\right)$ is generated online via the estimation of the parameters ${\tilde{\alpha }}_{g}$. In order to implement this controller, additional precautions have to be made to guarantee that ${\tilde{\alpha }}_{g}$ remains in a feasible region in which $\hat{G}\left(x,{\tilde{\alpha }}_{g}\right)$ is regular. Therefore, we modify the sliding mode control term (38) as follows [9]:

(39) usG^x,α~gε0Ip+G^x,α~gG^Tx,α~g−1·−F^x,α~f+v+K0sgn⁡s,

where *ε*_0_ is a small positive constant.

Within the sliding mode control term (39), we have used the regularized inverse of ${\hat{G}}^{-1}\left(x,{\tilde{\alpha }}_{g}\right)$ defined as

(40) $$\begin{array}{c} \hat{G}\left(\;x,{\tilde{\alpha }}_{g}\;\right){\left[\;\varepsilon_{0}I_{p}+\hat{G}\left(\;x,{\tilde{\alpha }}_{g}\;\right){\hat{G}}^{T}\left(\;x,{\tilde{\alpha }}_{g}\;\right)\;\right]}^{-1}. \end{array}$$

In fact, the regularized inverse (40) is well-defined even when $\hat{G}\left(x,{\tilde{\alpha }}_{g}\right)$ is singular, and the sliding mode control term (38) is always well-defined.

Even though the control law (39) is always well-defined, it cannot guarantee alone the stability of the closed loop system. It is due, partly, to the approximation of ${\hat{G}}^{-1}\left(x,{\tilde{\alpha }}_{g}\right)$ by the regularized inverse and, partly, to the unavoidable reconstruction errors of the unknown functions *F*(*x*) and *G*(*x*). For these reasons, and hoping for the cancellation of these approximations errors, we append to the controller (39) a robustifying control term *u*_*r*_ [8]

(41) $$\begin{array}{c} u=u_{s}+u_{r}. \end{array}$$

The controller (41) is the sum of two control terms: a modified sliding mode control term, *u*_*s*_

(42) usG^x,α~gε0Ip+G^x,α~gG^Tx,α~g−1·−F^x,α~f+v+K0sgn⁡s,

and a robustifying control term, *u*_*r*_

(43) $$\begin{array}{c} u_{r}=\frac{s\left|s^{T}\right|\left({\overset{-}{\varepsilon }}_{f}+{\overset{-}{\varepsilon }}_{g}\left|u_{s}\right|+\left|u_{0}\right|\right)}{\sigma_{0}{\left\|s\right\|}^{2}+\delta }, \end{array}$$

where *u*_0_ is

(44) u0ε0ε0Ip+G^x,α~gG^Tx,α~g−1·−F^x,α~f+v+K0sgn⁡s

and *δ* is a design time-varying parameter defined below.

In order to meet the control objectives, the adaptive parameters ${\tilde{\alpha }}_{fi}$, ${\tilde{\alpha }}_{gij}$, and the design parameter *δ* are updated by the following adaptive laws:

(45) α~˙fi=−ηfiξfixsi,

(46) α~˙gij=−ηgijξgijxsiusi,

(47) δ˙=−η0sTε−f+ε−gus+u0σ0s2+δ,

where *η*_*fi*_, *η*_*gij*_, *η*_0_, *δ*(0) > 0.

Then, we can prove the following theorem.

**Theorem 3 (consider system (1)).**

Suppose that Assumptions 1 and 2 are satisfied. Then the control law defined by (41) and (42), with adaptation laws given by (45)–(47), guarantees the following properties:

(3.1) All signals in the closed loop system are bounded.
(3.2) The tracking errors and its derivatives decrease asymptotically to zero; that is, *e*_*i*_^*j*^(*t*) → 0 as *t* → *∞* for *i* = 1,…, *p* and *j* = 0,1,…, *r*_*i*_ − 1.

**Proof**

Using the control law (51), (10) can be rewritten as

(48) s˙=v−Fx−Gx−G^x,α~guc−G^x,α~gus−Gxur.

By introducing the control term (42)–(48), we obtain

(49) s˙=−K0sgn⁡s−Fx−F^x,α~f−Gx−G^x,α~gus+u0−Gxur.

Here, we have used the fact that

(50) G^x,α~gG^Tx,α~gε0Ip+G^x,α~gG^Tx,α~g−1=Ip−ε0ε0Ip+G^x,α~gG^Tx,α~g−1.

From (37), one can write (49) as

(51) s˙=−K0sgn⁡s−F^x,α~f∗−F^x,α~f−G^x,α~g∗−G^x,α~gus+u0−Gxur+u0−εfx−εgxus.

Multiplying *s*^*T*^ to (51) gives

(52) sTs˙=−sTK0sgn⁡s−∑i=1pξfiTxα~−fisi−∑i=1p∑j=1pξgijTxα~−gijsiusj−sTGxur+sTu0−sTεfx−sTεgxus.

Let us now consider the following Lyapunov function candidate:

(53) V=12sTs+12∑i=1p1ηfiα~−fiTα~−fi+12∑i=1p∑j=1p1ηgijα~−gijTα~−gij+12η0δ2,

whose time derivative is given by

(54) $$\begin{array}{c} \dot{V}=s^{T}\dot{s}-\overset{p}{\underset{i=1}{\sum }}\frac{1}{\eta_{fi}}{\overset{-}{\tilde{\alpha }}}_{fi}^{T}{\dot{\overset{-}{\tilde{\alpha }}}}_{fi}-\overset{p}{\underset{i=1}{\sum }}\overset{p}{\underset{j=1}{\sum }}\frac{1}{\eta_{gij}}{\overset{-}{\tilde{\alpha }}}_{gij}^{T}{\dot{\overset{-}{\tilde{\alpha }}}}_{gij}+\frac{1}{\eta_{0}}\delta \dot{\delta }. \end{array}$$

With (52), (53) can be expressed as

(55) $$\begin{array}{c} \dot{V}=s^{T}K_{0}\mathrm{sgn}\left(\;s\;\right)+{\dot{V}}_{1}+{\dot{V}}_{2}, \end{array}$$

where

(56) V˙1=−∑i=1pα~−fiTξfixsi+1ηfiα~−˙fi−∑i=1p∑j=1pα~−gijTξgijxsiusj+1ηgijα~−˙gij,

(57) V˙2=−sTGxur+sTu0−sTεfx−sTεgxus+1η0δδ˙.

Substituting the parameter adaptive laws (45) and (46) into (56) gives

(58) $$\begin{array}{c} {\dot{V}}_{1}=0. \end{array}$$

Using (43), we can write

(59) sTGxur≥sT·ε−f+ε−gus+u0−sTε−f+ε−gus+u0σ0s2+δ.

Here, we have used the inequality

(60) $$\begin{array}{c} s^{T}G\left(\;x\;\right)s\geq \sigma_{0}{\left\|\;s\;\right\|}^{2}, \end{array}$$

which is true because *G*(*x*) is assumed positive definite and satisfies *G*(*x*) ≥ *σ*_0_*I*_*p*_.

Equation (57) can be bounded as follows:

(61) $$\begin{array}{c} {\dot{V}}_{2}\leq -s^{T}G\left(\;x\;\right)u_{r}+\left|\;s^{T}\;\right|\left(\;{\overset{-}{\varepsilon }}_{f}+{\overset{-}{\varepsilon }}_{g}\left|\;u_{s}\;\right|+\left|\;u_{0}\;\right|\;\right)+\frac{1}{\eta_{0}}\delta \dot{\delta }. \end{array}$$

With (60), (61) becomes

(62) $$\begin{array}{c} {\dot{V}}_{2}\leq -\frac{\delta \left|s^{T}\right|\left({\overset{-}{\varepsilon }}_{f}+{\overset{-}{\varepsilon }}_{g}\left|u_{s}\right|+\left|u_{0}\right|\right)}{\sigma_{0}{\left\|s\right\|}^{2}+\delta }+\frac{1}{\eta_{0}}\delta \dot{\delta }. \end{array}$$

Using (47) in (62) yields

(63) $$\begin{array}{c} {\dot{V}}_{2}\leq 0. \end{array}$$

From (58) and (63), it follows that

(64) $$\begin{array}{c} \dot{V}\leq -s^{T}K_{0}\mathrm{sgn}\left(\;s\;\right)=-\overset{p}{\underset{i=1}{\sum }}k_{oi}\left|\;s_{i}\;\right|. \end{array}$$

By Barbalat's lemma [5], it can conclude that *s* → 0 as *t* → *∞*. In spite of the demonstrated properties of the controller, the hitting control law leads to the well-known chattering phenomenon. In order to overcome the undesirable chattering effects, the sign function is replaced with the saturation function [5].

## 5. Simulation Results

**Figure 3:**
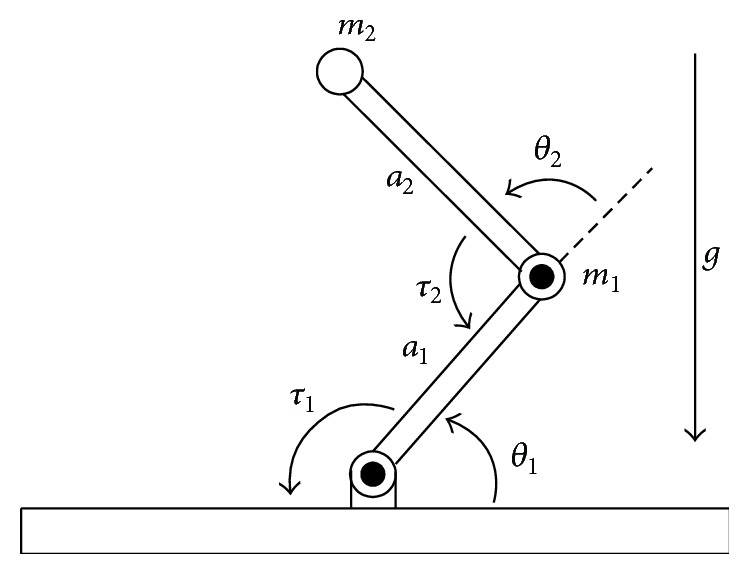
Planar model of the two-link manipulator.

In this section, we test the AIT2FSMC design on the tracking control of a two-link robot. Consider a two-link rigid robot manipulator moving a horizontal plant in Figure 3. The first link is mounted on a rigid base by means of frictionless hinges and the second is mounted at the end of first link by means of a frictionless ball bearing. The dynamic equations of this MIMO system are given by [5]

(65) q¨1q¨2M11M12M21M22−1·u1u2−−hq˙2−hq˙1+q˙2hq˙10q˙1q˙2,

where

(66) M11=a1+2a3cos⁡q2+2a4sin⁡q2,M22=a2,M12=M21=a2+a3cos⁡q2+a4sin⁡q2h=a3sinq2−a4cosq2,

with

(67) a1=I1+m1lc12+Ie+melce2+mel12,a2=Ie+melce2,a3=mel1lcecos⁡δe,a4=mel1lcesin⁡δe.

In the simulation, the following parameter values are used:

(68) m1=1,me=2,l1=1,lcl=0.5,lce=0.6,I1=0.12,Ie=0.25,δe=30°.

Let $y={\left[\begin{array}{cc} y_{1} & y_{2} \end{array}\right]}^{T}={\left[\begin{array}{cc} q_{1} & q_{2} \end{array}\right]}^{T}$, $u={\left[\begin{array}{cc} u_{1} & u_{2} \end{array}\right]}^{T}$, $x={\left[\begin{array}{cccc} q_{1} & {\dot{q}}_{1} & q_{2} & {\dot{q}}_{2} \end{array}\right]}^{T}$, and

(69) Fx=f1xf2x=−M−hq˙2−hq˙1+q˙2hq˙10q˙1q˙2,Gx=g11xg12xg21xg22x=M−1=M11M12M21M22,

and then, the robot system (65) can be described as follows:

(70) $$\begin{array}{c} \ddot{y}=F\left(\;x\;\right)+G\left(\;x\;\right)u, \end{array}$$

which is the input-output form given by (3). Since the matrix *M* is positive definite [5], then it is always regular and *G*(*x*) = *M*^−1^ is positive definite.

The control objective is to force the system outputs *q*_1_ and *q*_2_ to track the sinusoidal desired trajectories *y*_*d*1_ = sin⁡(*t*) and *y*_*d*2_ = sin⁡(*t*). In order to analyze the performance of the AIT2FSMC, we compared the AIT2FSMC with the A-Fuzzy Sliding Mode Controller (AFSMC) which used the type-1 FLS to approximate the nonlinear *F*(*x*) and *G*(*x*). The external disturbances ${\left[\begin{array}{cc} \cos\left(t\right) & \sin\left(t\right) \end{array}\right]}^{T}$are added to system (65). Since the components of *F*(*x*) and *G*(*x*) are assumed unknown, two fuzzy systems in the form of (30) are used to approximate the elements of *F*(*x*), and four are used to approximate the elements of *G*(*x*). In the AIT2FSMC and the AFSMC, the sliding surface is selected as with *λ*_1_, *λ*_2_ = 5 and the design parameters used in this simulation are chosen as follows: *K*_0_ = 0.3*I*_2_, *ε*_0_ = 0.1, *η*_*fi*_ = 0.5, *η*_*gij*_ = 0.5 for *i*, *j* = 2 and the initial conditions of robot are selected as $x(0)=\left[\begin{array}{cccc} 0.5 & 0 & 0.25 & 0 \end{array}\right]$. The fuzzy systems used to describe *F*(*x*) have *q*_1_(*t*), ${\dot{q}}_{1}(t)$, *q*_2_(*t*), and ${\dot{q}}_{2}(t)$ as inputs. The input membership functions and parameters for the AIT2FSMC and AFSMC are shown in Table 1.

**Figure 4:**
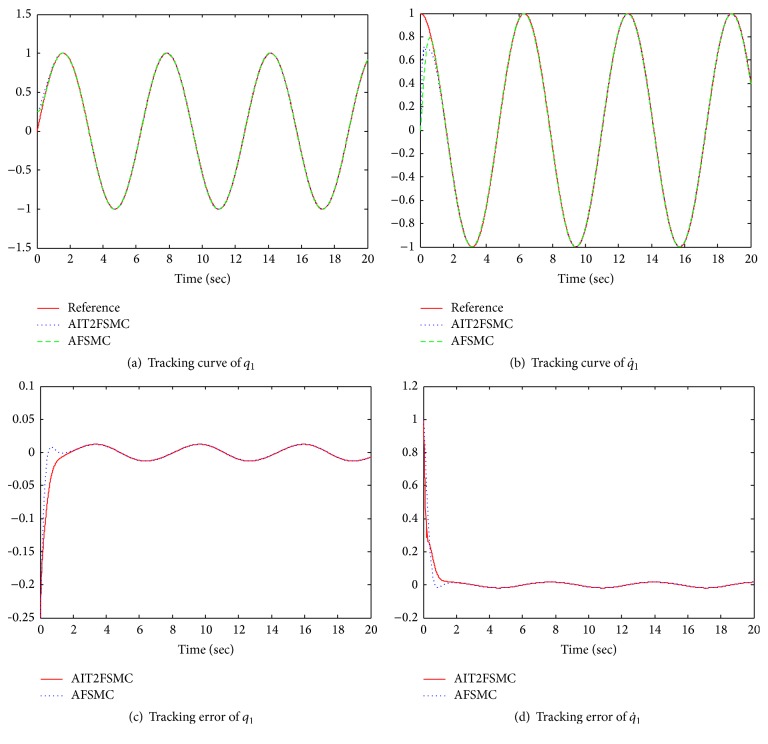
Tracking results of link 1.

**Figure 5:**
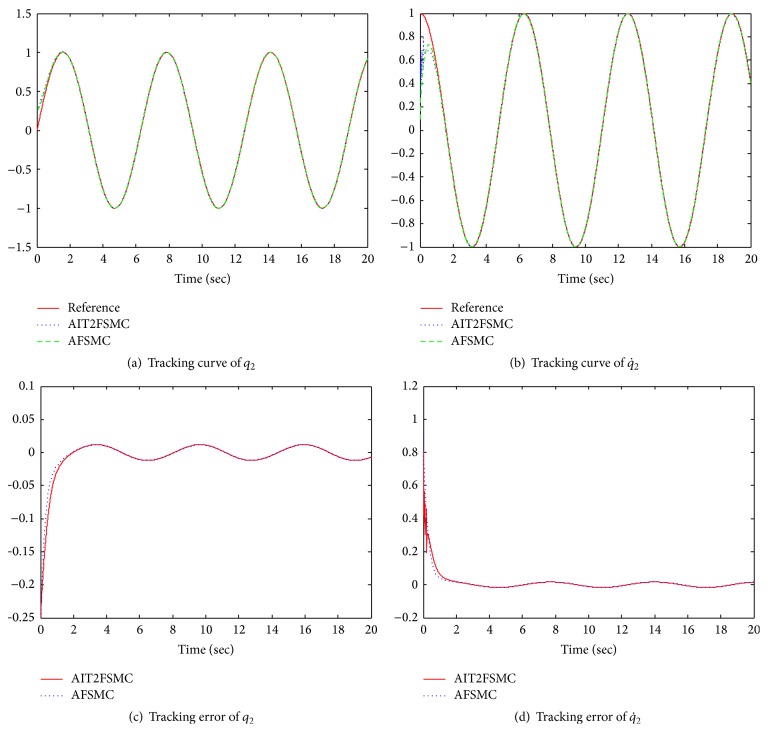
Tracking results of link 2.

As shown in Figures 4 and 5, the AIT2FSMC shows the better performance than the AFSMC. In the AFSMC, a type-1 FLS, which is not able to handle rule uncertainties, is used for control of unknown nonlinear MIMO system. Therefore, the system performance is deteriorated by the disturbance. Meanwhile, the proposed AIT2FSMC utilizes the interval type-2 FLS. The simulation results show that the interval type-2 FLS is able to handle rule uncertainties, and thus the system performance is compensated by the interval type-2 FLS [13].

## 6. Discussion

In this paper, we propose a novel advanced interval type-2 fuzzy sliding mode control (AIT2FSMC) for a class of uncertain nonlinear MIMO systems with external disturbances. The parameters of the proposed AIT2FSMC system, as well as the approximation error bound, are tuned online. The control laws are obtained in the Lyapunov sense to ensure the stability of the control system.

Unlike the conventional SMCs, the design of the proposed AIT2FSMC is independent of the mathematical model of the system and can be applied to both unknown and uncertain nonlinear MIMO systems. Furthermore, the uncertainty bound is not needed to be available beforehand. Simulation results performed on a two-link robot manipulator demonstrate the feasibility of the proposed control system.

## Figures and Tables

**Table 1:** The parameters of input membership functions for the AIT2FSMC and the AFSMC.

	Negative			Zero			Positive		
	*m*_*N*1_	*m*_*N*2_	*σ*_*N*_	*m*_*Z*1_	*m*_*Z*2_	*σ*_*Z*_	*m*_*P*1_	*m*_*P*2_	*σ*_*P*_
AIT2FSMC	−1.15	−0.35	0.6	−0.01	0.01	0.6	1.15	1.35	0.6
AFSMC	−1.25	−1.25	0.6	0	0	0.6	1.25	1.25	0.6
